# Evaluation of multiple consensus criteria for autoimmune encephalitis and temporal analysis of symptoms in a pediatric encephalitis cohort

**DOI:** 10.3389/fneur.2022.952317

**Published:** 2022-09-27

**Authors:** Tiffany Pointon, Ryan Ward, Anusha Yeshokumar, Amanda Piquet, Teri Schreiner, Ryan Kammeyer

**Affiliations:** ^1^Section of Child Neurology, Department of Pediatrics, University of Colorado School of Medicine, Aurora, CO, United States; ^2^School of Medicine, University of Colorado, Aurora, CO, United States; ^3^Department of Neurology, Icahn School of Medicine at Mount Sinai, New York, NY, United States; ^4^Section of Neuroimmunology, Department of Neurology, University of Colorado School of Medicine, Aurora, CO, United States

**Keywords:** autoimmune encephalitis, infectious encephalitis, neuroimmune disease, pediatric neurology, diagnostic criteria, temporal analysis and evaluation

## Abstract

**Objective:**

To evaluate the sensitivity and specificity of current criteria for the diagnosis of autoimmune encephalitis (AE) and the temporal onset of neuropsychiatric symptoms (NP) in a pediatric encephalitis cohort.

**Background:**

Multiple criteria for AE have been developed, including the Graus and pediatric-focused Cellucci consensus criteria, and the Determining Etiology in Encephalitis (DEE) score for patients with encephalitis. Early identification and treatment of AE is crucial to improve outcomes, but this can be difficult given the frequent overlap of clinical presentation between AE and infectious encephalitis (IE).

**Design/methods:**

A retrospective review was conducted of patients seen at our institution from 2000 to 2021 with a final diagnosis of AE or IE. These were narrowed through multiple exclusions to etiology-confirmed IE or antibody-positive/negative AE. Time of onset or results of all symptoms and diagnostics were recorded. Sensitivity and specificity of each criterion under various clinical scenarios were calculated over the first month after initial NP symptom onset.

**Results:**

A total of 23 antibody-positive AE, 9 antibody-negative AE and 23 IE patients were included in final analysis. Under an idealized scenario with rapid initial diagnostic evaluations, the sensitivity for pediatric AE by day 28 after onset of NP symptoms approached 90% for both Cellucci and Graus criteria. Specificity within these 28 days was low without infectious testing results, increasing the greatest with rapid PCR testing and second with infectious antibody testing—reaching ~90% with both. A DEE score of 3 provided a specificity of 100% in identifying IE, but low sensitivity (29%). Symptoms were noted to cluster within several days of onset in IE, but in AE were spread out. Personality/behavioral change, speech change, affective disorder, and sleep disturbance were noted more often in AE, while fever, elevated C-reactive protein or CSF protein, and abnormal MRI-Brain occurred more often in IE.

**Conclusion:**

In this study, we provide the first evaluation of the Cellucci criteria and the first validation of the DEE score in the differentiation of pediatric AE and IE. Further refinement of AE criteria is needed to improve early detection and treatment of pediatric AE.

## Introduction

Autoimmune encephalitis (AE) occurs when the immune system is misdirected into attacking the brain, resulting in neuroinflammation and neurologic dysfunction. Anti-N-methyl-D-aspartate receptor encephalitis (anti-NMDARE) is the most common cause of AE in children and young adults, and may be more common than individual viral etiologies in encephalitis with negative initial infectious testing (1). Antibody-negative AE is seen in 11–52% of total cases of pediatric AE (2–4), and represents a particular challenge in diagnosis (5). Early initiation of immunotherapy [within the first month of onset of neuropsychiatric (NP) symptoms] has been shown to decrease relapse rates and improve long term outcomes, highlighting the critical importance of timely recognition and diagnosis of AE (6–8).

To assist in distinguishing AE from other causes of encephalopathy, behavior/psychiatric changes, seizures, or infectious encephalitis (IE), several clinical consensus criteria have been developed. The Graus criteria are the most well-known criteria for AE, with separate clinical/paraclinical criteria for possible AE, probable or definite anti-NMDARE, and probable autoantibody-negative AE (9). Pediatric-specific criteria have since been proposed for the diagnosis of AE by Cellucci et al. to reflect the broader range of presenting symptoms and diagnostic challenges specific to pediatric AE (5), achieving appropriate sensitivity and specificity in general cohorts of suspected pediatric AE (10). Recently, the Determining Etiology in Encephalitis (DEE) score has been developed for differentiation of AE and IE in encephalitis (11). While the clinical criteria have guided clinicians in an earlier recognition of AE and earlier immunotherapy initiation, they are time dependent (i.e., the accuracy of the criteria increase over time as symptoms accrue and become more severe), causing tension between initiation of early empiric immunotherapy for AE and ensuring a comprehensive exclusion of infectious etiologies before doing so. Prior studies have shown that the specificity of Graus' possible AE criteria in cohorts of encephalitis may be as low as 8% prior to infectious testing results being known (12). The added contribution to specificity from each phase of infectious testing—polymerase chain reaction (PCR—rapid), antibody (days), and biopsy—have not been assessed.

In our study, we provide the first evaluation of the Cellucci pediatric AE criteria and the DEE score in the difficult clinical scenario of differentiating between AE and IE. For comparison, we evaluated the Graus possible AE and anti-NMDARE criteria in scenarios without diagnostic testing, with rapid diagnostic testing, and with real-world results. We also assessed the contribution of individual infectious testing modalities to specificity. We focused on the first month after initial NP symptom onset given the importance of this early time period for long-term outcome. Finally, we compared onset of symptoms and diagnostics between AE and IE, to provide recommendations for future refinement of clinical criteria in the early differentiation of pediatric AE and IE.

## Methods

### Patient selection

We conducted a retrospective review of pediatric patients seen at Children's Hospital Colorado between January 2000 and February 2021.

**Figure 1:**
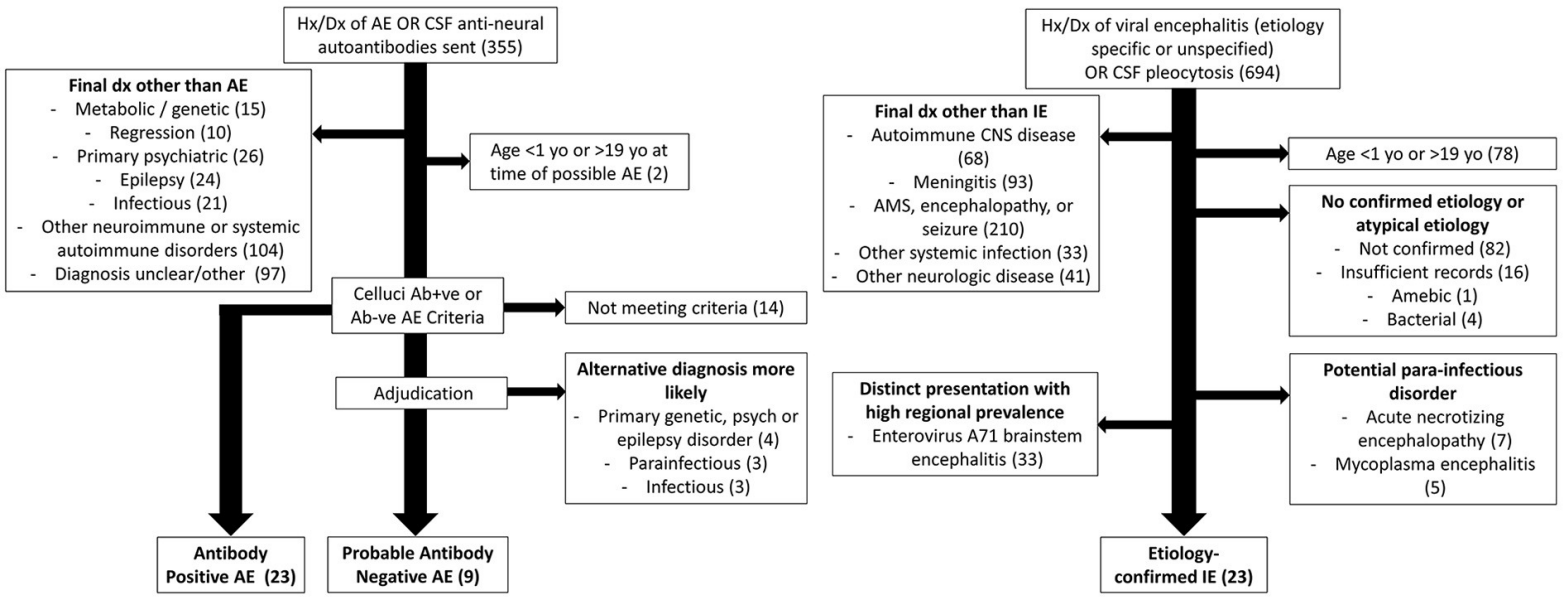
Inclusion and exclusion of patients with AE or IE.

Patients with possible AE were initially identified through an electronic medical record (EMR) query using Slicer Dicer (Epic Systems) for patients with a diagnosis or medical history under the broad diagnostic group code (DGC) of autoimmune encephalitis (encompasses 87 related ICD-10 codes), or on whom cerebrospinal fluid (CSF) anti-neuronal autoantibodies had been sent (Figure 1). After individual chart review, patients with a final diagnosis of AE by a physician at the last clinical encounter and an age of ≥1 years-old and ≤ 19 years-old at the time of initial NP symptoms were included in subsequent evaluation. These patients were categorized as having a diagnosis of antibody-positive AE if meeting the pediatric AE criteria for this disorder as proposed by Cellucci et al. (5). Patients meeting the Cellucci criteria for probable antibody-negative AE subsequently underwent adjudication by author AY, who had not previously been involved in their clinical care. AY was provided all results of blood, CSF, magnetic resonance imaging (MRI), electroencephalography (EEG), and other diagnostic testing, presenting symptoms and time course, response of symptoms to immunotherapy treatment, and any other relevant objective clinical information. Clinical notes were not provided to minimize subjective data and impressions from the treating physicians. Patients with known recent preceding or concurrent IE were not included, given potential overlap of symptom and diagnostic testing.

Patients with potential IE were initially identified through an EMR query for patients with a diagnosis or medical history under the DGC of viral encephalitis (encompasses 324 related ICD-10 codes), or from review under secondary use of previously collected cases of meningitis, encephalitis, and CSF pleocytosis (Figure 1). Patients who had a final diagnosis of IE at the last clinical encounter and an age of ≥1 years-old and ≤ 19 years-old at the time of initial NP symptoms were included in subsequent evaluation. A lack of confirmed infectious etiology was cause for exclusion given the uncertainty of whether these cases represented a direct infectious process vs. a para-infectious or unrecognized primary autoimmune process. Mycoplasma may cause encephalitis through an indirect autoimmune or para-infectious process and was therefore excluded (13). Both acute necrotizing encephalopathy (ANE) and enterovirus A71 have unique clinical or radiographic features that allow for ready distinction from pediatric AE, and so were also excluded (14). All etiology-confirmed IE that was included in final analysis met the International Encephalitis Consortium's diagnostic criteria for confirmed encephalitis (15).

After completion of chart review and exclusion of patients with an alternative diagnosis or uncertain encephalitis etiology, patients with antibody-positive AE, antibody-negative AE, or etiology confirmed IE were included in final analysis.

### Data collection

Detailed clinical and paraclinical data were obtained through chart review and input into a standardized REDCap data collection tool for all patients included in final analysis. Demographic information, medical history, constitutional and NP symptoms, and paraclinical data were recorded, as well as the time of presentation or test result for each symptom or diagnostic test. NP symptoms included any NP symptoms or signs noted by the patient, caregiver, or medical provider, with the day of onset of NP symptoms being recorded as Day 0 of the illness. NP symptoms were categorized as detailed in prior clinical consensus criteria (5, 9). Constitutional symptoms included any non-neuropsychiatric symptoms occurring within the month prior to or after the onset of NP symptoms. Paraclinical data included serum and CSF inflammatory and infectious studies [including serum C-reactive protein (CRP) and erythrocyte sedimentation rate (ESR)], EEG, MRI of the brain (MRI-Brain), and oncologic testing as applicable.

### Clinical consensus criteria for AE and DEE score

Several consensus criteria for AE and the DEE score were applied in our analysis. The Graus clinical consensus criteria for AE were published in 2016 (9). Specific criteria used in our analyses included the possible AE criteria and the probable NMDARE criteria. The probable NMDARE criteria is solely symptom-based, while the possible AE criteria requires either multiple neurologic and psychiatric symptoms, or at least one NP symptom in addition to CSF or MRI evidence of neuroinflammation/encephalitis. The Cellucci criteria for pediatric AE were published in 2020, as a refinement of the Graus criteria for use in pediatric populations (5). There are three diagnostic categories present within this criteria—that of possible AE, probable antibody-negative AE, and definite antibody-positive AE—each of which are applied in our analyses. The Cellucci criteria differs from the Graus criteria in several ways, including the use of EEG findings in determining altered mental status, inclusion of developmental regression for younger children, an increased focus on symptoms relevant to the most common pediatric AE, NMDARE, and removal of CSF or MRI evidence of inflammation from the possible AE criteria. Each of these criteria require a rapid, subacute onset of symptoms over < 3 months, and a reasonable exclusion of other disorders (at the physician's discretion).

The Determining Etiology in Encephalitis (DEE) score was recently described to assist in distinguishing between AE and IE based on presence of fever, initial CSF cell count ≥50 cells/μL, and initial CSF protein ≥75 mg/dL—quickly and easily available clinical parameters (11). These parameters were evaluated for predictive value from a set of author-selected parameters thought to be clinically relevant. This score has not yet undergone validation with a separate cohort.

### Data analysis

For the temporal data collected, the published consensus criteria for the diagnosis of possible or probable AE were applied to each case of AE and IE to determine the day on which each patient met the AE criteria under various clinical scenarios (5, 9). To assess the criteria and local diagnostic testing alone, results of autoantibody testing were not included in the analysis. An important part of each criterion, the “exclusion of alternative etiologies,” was performed by including objective infectious testing. Given the interest in the early phase of encephalitis, we focused on the first 4 weeks (28 days) after NP symptom onset. The DEE score was calculated as previously described (11).

For sensitivity, the clinical scenarios of interest included “Symptoms Only” (prior to any diagnostic testing), “Initial Diagnostics” (all initial paraclinical testing completed—MRI, EEG, and basic CSF studies without autoantibodies—at the time they were performed clinically), and “Immediate Diagnostics” (all initial paraclinical testing, but with the assumption that the testing was performed immediately once they met criteria for possible AE by symptomatology alone). Temporal curves were developed for these scenarios for the Cellucci possible/probable AE criteria and the Graus criteria (both possible AE and probable NMDARE). These are meant to mirror three separate scenarios in which the clinical criteria may be applied for decision-making in clinical practice. First, when a patient initially presents and the provider must decide whether to order paraclinical testing for further evaluation based on symptoms alone; second, a real-world scenario after these initial paraclinical tests result (but autoantibodies have not yet returned) and the provide must decide whether to initiate empiric immunotherapy; and finally, an idealized scenario where testing is obtained immediately after meeting AE criteria by clinical symptoms alone, and all abnormalities seen on real world testing are also present at that early time point. This idealized scenario leads to an estimate of the maximal sensitivity for each full criterion (includes both clinical and paraclinical data).

For specificity, a set of temporal curves were created for three separate scenarios: at completion of all initial paraclinical testing alone, with the addition of infectious PCR testing, and with the addition of both infectious PCR and antibody testing. These correspond to scenarios in which a provider has obtained all initial paraclinical testing and must decide whether they have completed a “reasonable exclusion” of infectious causes before starting empiric immunotherapy (5, 9). These scenarios highlight both the relative contributions of each of these infectious diagnostic modalities to improving the specificity and certainty of the criteria, as well as delays in treatment that may occur while waiting for this testing to return.

### Statistical analysis

Descriptive statistics were used for demographic, admission, and treatment variables. The proportions for the presence of relevant clinical or paraclinical features were compared between the AE and IE cohorts using a Chi-squared or Fisher exact test. Given the non-normal distribution of the temporal data, the medians for continuous variables were compared using Wilcoxon rank sum tests. Given the number of potential comparisons, only those comparisons of interest were performed, and a Bonferroni correction was performed for an original α = 0.05, giving an adjusted-α = 0.0013. For calculations of sensitivity, specificity, and accuracy, true positives were patients with AE meeting the specified criteria for AE in a given clinical scenario at that time point, false positives were patients with IE meeting the specified AE criteria, true negatives were patients with IE not meeting the specified AE criteria, and false negatives were patients with AE not meeting the specified AE criteria.

### Standard protocol approvals, registrations, and patient consents

Approval to conduct this secondary retrospective analysis was received from the Colorado Multiple Institutional Review Board (COMIRB). Participant consent was not required by the board under exempt status.

## Results

### Patient inclusion, demographics, and descriptive statistics

A total of 23 patients with antibody-positive AE, 9 patients with antibody-negative AE, and 23 patients with etiology-confirmed IE were included in final analysis, as shown in Figure 1. Fourteen patients with a final diagnosis of antibody-negative AE by the treating physician in chart review were excluded after failing to meet the Cellucci criteria. Four of these that were excluded by the Cellucci criteria were thought to be clinically consistent with antibody-negative AE by our author AY (these patients were not included in final analysis). Of the 19 patients categorized as antibody-negative AE after application of the Cellucci criteria, 10 were thought more likely to have a primary psychiatric disorder, epilepsy, genetic disorder, IE, or a para-infectious process after review of the case by the adjudicating author AY. These 10 patients were also not included in final analysis, given the conflicting diagnoses from AE criteria and blinded adjudication.

The demographics and final diagnoses of these groups are shown in Table 1. Among the antibody-positive AE group, the majority were anti-NMDARE, with one case of anti-glycine receptor (anti-Gly-R) encephalitis. Among IE cases, the majority were HSVE (39%) or West Nile Virus (WNV) encephalitis (30%). Immunotherapy was started a median of 3 and 2.5 days after patients had met the AE clinical criteria (including initial diagnostic testing and return of infectious testing) for antibody-positive and negative AE patients, respectively. Infectious testing resulting in the diagnosis of IE returned in a median of 1.5 days (PCR) and 4 days (infectious antibodies) after being sent.

**Table 1:** Demographics and descriptive statistics of patients included in study.

	**Autoimmune encephalitis**		**Infectious encephalitis**
	**Antibody positive**	**Antibody negative**	
Total, *N*	23	9	23
Age, years, median (range)	10.5 (2–17)	8 (2–14)	11 (2–19)
Gender, female, *n* (%)	18 (78)	5 (56)	8 (35)
**Race/ethnicity**, ***n*** **(%)**
White/caucasian	6 (25)	3 (33)	7 (30)
Hispanic	8 (33)	3 (33)	14 (61)
Black	2 (8)	1 (11)	2 (9)
Asian	1 (4)	0 (0)	0 (0)
Other	7 (29)	2 (22)	0 (0)
Encephalitis etiology, *n* (%)	Anti-NMDAR: 21 (91) Anti-NMDAR/Anti-MOG: 1 (4) Anti-Gly-R: 1 (4)	Antibody-negative: 9 (100)	HSV: 9 (39) WNV: 7 (30) HHV 6: 3 (13) EBV: 1 (4) VZV: 1 (4) HIV: 1 (4) Influenza: 1 (4)
Admission length, days, median (range)	26 (6–126)	14 (4–414)	10.5 (1–125)
ICU Admission, *n* (%)	8 (35)	5 (55)	14 (60)
Time until immunotherapy, days, median (range)	14 (1–72)	13 (4–71)	N/A

Anti-MOG, Anti-myelin oligodendrocyte glycoprotein; ICU, Intensive care unit; HHV6, Human herpes virus 6; EBV, Epstein-Barr virus; VZV, Varicella-zoster virus; HIV, Human immunodeficiency virus.

### Frequency and temporal onset of symptoms and diagnostic abnormalities

Differences between the clinical symptoms and paraclinical testing seen in our AE (combined antibody-positive and antibody-negative) and IE cohorts are shown in Table 2. Personality/behavioral change, speech change, affective disorder, movement disorder, and sleep disorders were seen more often in AE, while fever, elevated CRP, elevated CSF protein, and MRI abnormalities were seen more often in IE. Descriptive statistics of the frequency of clinical symptoms and paraclinical testing abnormalities for antibody-positive and antibody-negative AE individually is provided in the (Supplementary Table 1).

**Table 2:** Clinical symptoms and paraclinical diagnostic testing in AE and IE.

	**Autoimmune encephalitis**	**Infectious encephalitis**	***P*-value**
**Constitutional symptoms**, ***n*** **(%)**
Fever	8 (25)	21 (91)	< 0.0001*
Headache	11 (34)	13 (57)	0.10
Upper respiratory	9 (28)	9 (39)	0.39
Gastrointestinal	13 (41)	12 (52)	0.40
Myalgias	0 (0)	4 (17)	0.014
Rash	2 (6)	3 (13)	0.39
**Neuropsychiatric symptoms, n (%)**
Personality or behavioral change	29 (91)	2 (9)	< 0.0001*
Cognitive dysfunction or regression^a^	30 (94)	19 (82)	0.19
Speech change	29 (91)	6 (26)	< 0.0001*
Seizure	24 (75)	11 (48)	0.038
Psychosis	15 (47)	3 (13)	0.0083
Affective disorder	15 (47)	0 (0)	0.00012*
Dysautonomia	6 (19)	1 (4)	0.11
Movement disorder	17 (53)	3 (13)	0.0023
Insomnia/hypersomnia	16 (50)	1 (4)	0.0003*
Focal neuro deficit^b^	19 (59)	6 (26)	0.014
**Diagnostic abnormalities, n/no. tested (%)**
**EEG**
Any abnormality	32/32 (100)	19/19 (100)	n/a
Background slowing (generalized or focal)	25 (78)	15 (79)	0.94
Epileptiform discharges	11 (34)	7 (32)	0.86
Extreme delta brush	3 (9)	0 (0)	0.17
**MRI-brain**
Any abnormality	13/32 (41)	20/22 (91)	0.0002*
Meningeal enhancement	4 (13)	4 (18)	0.56
T2 hyperintensity, uni/bilateral temporal lobe	2 (6)	5 (23)	0.077
T2 hyperintensity, other grey/white matter	5 (16)	13 (59)	0.0009*
Diffusion restriction (not related to seizures)	0 (0)	3 (14)	0.032
**CSF** ^**c**^
Pleocytosis, >5 cells/μL	16/31 (52)	16/20 (73)	0.040
Pleocytosis, >20 cells/μL	7/31 (23)	12/20 (54)	0.007
Elevated protein, >45 mg/dL	0/31 (0)	7/20 (35)	0.0004*
Oligoclonal bands (≥2, CSF, unique)	12/21 (57)	0/4 (0)	0.035
**Blood**
Elevated CRP	1/27 (4)	8/16 (50)	0.0003*
Elevated ESR	3/23 (13)	8/14 (57)	0.0044
**Time until clinical criteria met**
Symptoms only, days, median (range)	Graus: 5 (0–52); Cellucci: 1 (0-52)	Graus: 0 (0–3); Cellucci: 0 (0–3)	0.013, AE Graus vs. Cellucci
Full criteria, days, median (range)	Graus: 8 (1–67); Cellucci: 11 (1–72)	Graus: 0.5 (0–3); Cellucci: 2 (0–9)	0.50, AE Graus vs. Cellucci

^*^indicates a *p*-value < the adj-α of 0.0013; ^a^The symptom “cognitive dysfunction or regression” included those with cognitive dysfunction, altered mental status, memory change, or regression; ^b^ Focal neurologic deficits included ataxia/gait imbalance, focal weakness, or cranial nerve palsies. ^c^Traumatic CSF (>1,000 red blood cells/μL) was excluded for analysis of cell count and protein.

**Figure 2:**
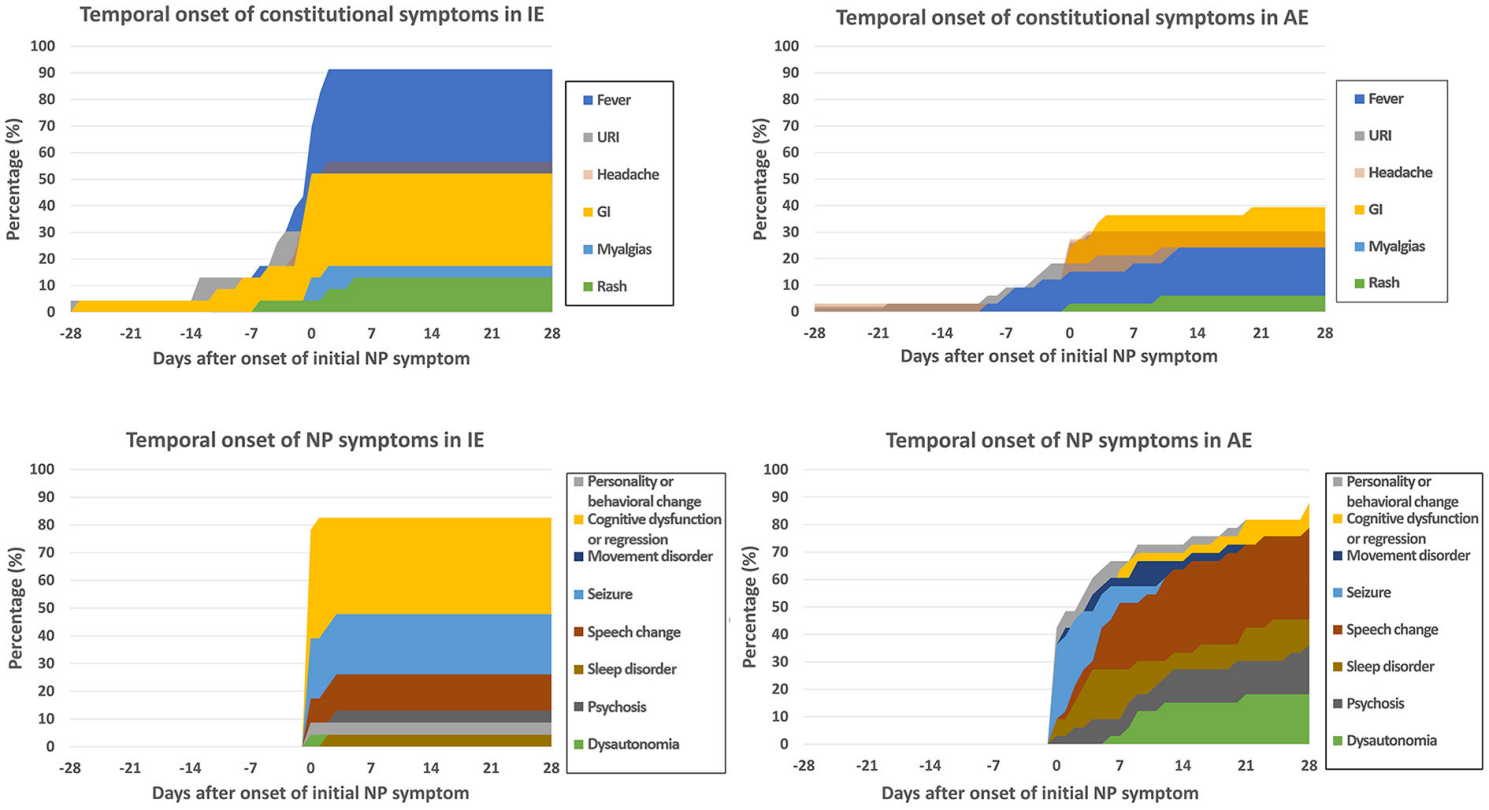
Temporal onset of constitutional and NP symptoms in IE and AE. Graphs present cumulative percentage of patients with each individual symptom relative to onset of initial NP symptom (Day 0).

The temporal onset of multiple NP symptoms in IE occurs over a shorter period compared to AE, as shown visually in Figure 2. A total of 13 IE cases (57%) never met Symptoms-Only Graus possible AE criteria, and 7 IE cases (30%) never met Symptoms-Only Cellucci possible AE criteria. These cases presented with seizure or altered mentation/cognition alone, without other NP symptoms. Of the remaining IE cases that did meet Symptoms-Only Cellucci or Graus criteria at one time point, all met this by day 3 after initial NP symptom onset—i.e., there was no longer than 3 days between the initial NP symptom and the development of additional NP symptoms.

### Sensitivity and specificity of clinical criteria

**Figure 3:**
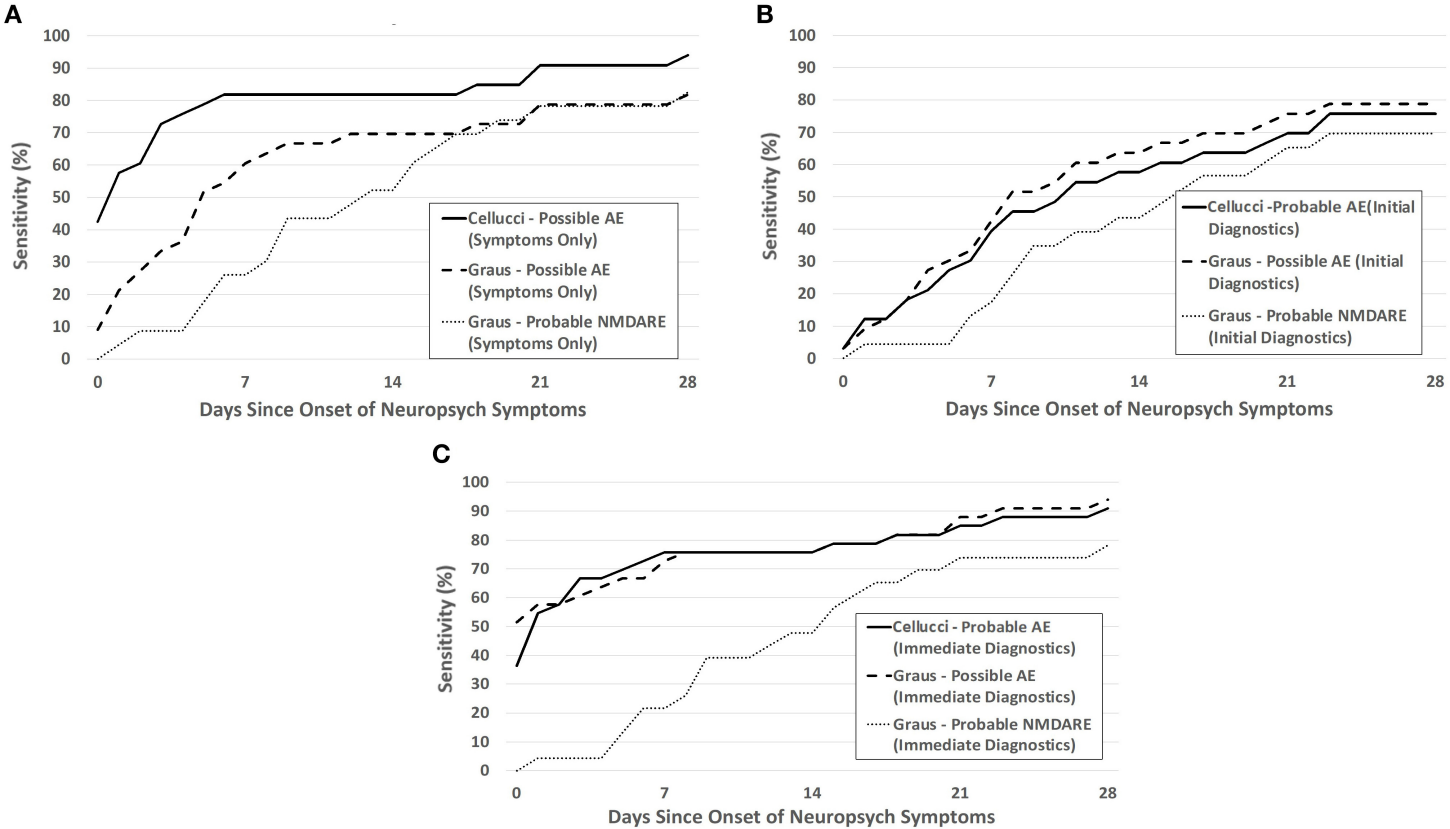
Sensitivity of the Cellucci and Graus criteria for pediatric AE over the first month after NP symptom onset utilizing **(A)** clinical symptoms alone (Symptoms Only), **(B)** both clinical symptoms and initial paraclinical diagnostic testing (EEG, MRI, CSF) without autoantibodies (Initial Diagnostics), and **(C)** assuming an idealized scenario where diagnostic testing are immediately obtained after meeting symptom criteria (Immediate Diagnostics). The probable NMDARE criteria was applied only to patients with NMDARE.

**Figure 4:**
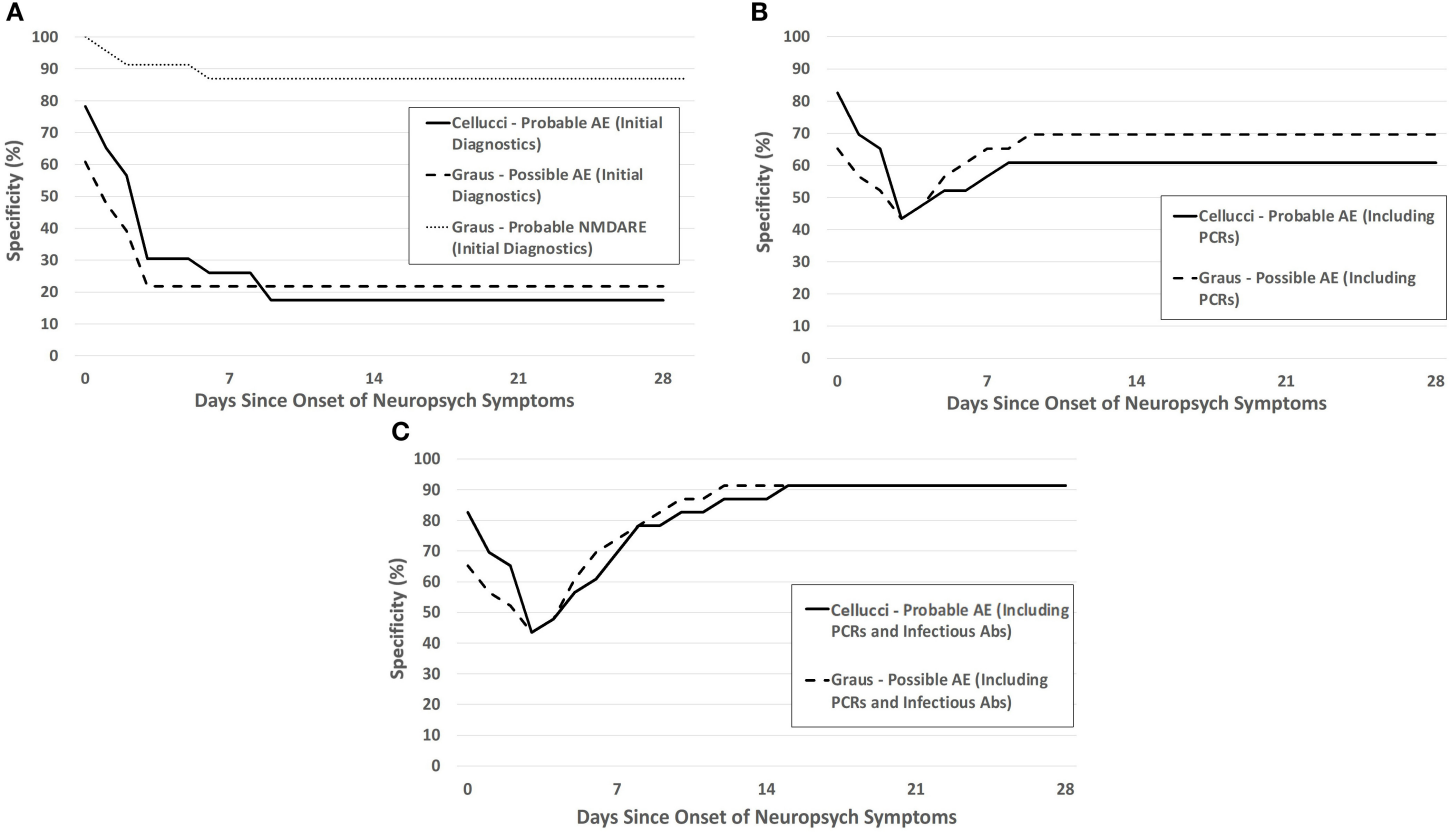
Specificity of the Cellucci and Graus criteria for pediatric AE over the first month after neuropsychiatric symptom onset with **(A)** clinical symptoms and initial diagnostic testing (without infectious PCRs or antibodies), **(B)** clinical and initial diagnostics including PCR testing, **(C)** clinical and initial diagnostics including both PCR testing and infectious antibody testing.

The sensitivity and specificity of the Cellucci and Graus criteria within the pediatric encephalitis cohort (AE and IE) are shown in Figures 3,4. Additional figures showing the sensitivity of these criteria within antibody-positive and antibody-negative AE, individually, is provided in the (Supplementary Figures 1, 2).

For clinical symptoms only, sensitivity was highest with use of the Cellucci possible AE criteria, as shown in Figure 3A. Sensitivity was lowered by the inclusion of real-time paraclinical testing (Initial Diagnostics), likely due to the delay in obtaining this testing (Figure 3B). With assumption of the idealized paraclinical testing (Immediate Diagnostics), both Graus and Cellucci performed very similarly, reaching ~90% sensitivity by day 28 (Figure 3C). There was no statistically significant difference between the accuracy of these two criteria at 28 days under the scenarios of Symptoms Only (61.8 vs. 56.4%, *p* = 0.56), Initial Diagnostics (50.9 vs. 60.0%, *p* = 0.34), or Initial Diagnostics including infectious PCR/antibodies (81.8 vs. 83.6%, *p* = 0.80). For patients with NMDARE, specificity was higher and sensitivity was lower when applying the Graus symptom-based probable NMDARE criteria than either general possible AE criteria—over the entire study period (extending past 28 days) the sensitivity and specificity of the probable NMDARE criteria each reached 87%.

Without any infectious-specific diagnostic testing, the specificity of each criterion (both Symptoms Only and with Initial Diagnostics) were low (Figure 4A). The specificity of the criteria increased with the addition of each infectious diagnostic modality—PCR testing provided the greatest increase in specificity, followed by infectious antibody testing (Figures 4B,C). The remainder of the IE patients were diagnosed by biopsy.

A total of 6 of 21 patients with IE and 0 of 32 patients with AE had a DEE score of 3 (presence of fever, initial CSF cell count ≥50 cells/μL, and initial CSF protein ≥75 mg/dL), giving a sensitivity of this score for AE being “highly unlikely” (i.e., of IE being likely) of 29%, and a specificity of 100%.

## Discussion

In this study, we provide an evaluation of the Cellucci criteria and DEE score in the differentiation of pediatric AE and IE, and identify temporal and clinical factors that may assist in the refinement of future criteria.

### Sensitivity and specificity of clinical criteria

Though sensitivity should be maximized during the first month after NP symptom onset, given the known association of delayed diagnosis >1 month with a worse clinical outcome, we found the sensitivity of both criteria at 28 days only reached ~80% in a real-world scenario and 90% in an ideal scenario. Approximately 10–20% of patients with pediatric AE would therefore be diagnosed outside the optimal 1-month window for immunotherapy if decision-making followed the criteria strictly. The real-world sensitivity is dependent on when providers decide to perform paraclinical testing; therefore, lower sensitivity may be due to a lack of recognition of symptoms of AE in children or early misdiagnosis. The use of the Symptoms Only clinical criteria gave sensitivities at 28 days of 81% (Graus) or 94% (Cellucci), suggesting the utility of these criteria in the decision to pursue further diagnostic testing for AE.

The inclusion of radiologic and intrathecal markers of inflammation in the clinical criteria are appropriate to help distinguish between inflammatory and non-inflammatory etiologies of symptoms. However, different forms of encephalitis are difficult to distinguish between by using readily available markers of inflammation, and so the clinical consensus criteria for AE also have difficulty with distinguishing between the two unless a “reasonable exclusion” of infection is made. Our study demonstrated the value of each phase of infectious testing for this “reasonable exclusion”—with initial rapid PCR testing increasing specificity of the clinical criteria from ~20% to 60–70%, and infectious antibodies (primarily CSF WNV IgM) increasing this further to 91%. The magnitude of the increase in the specificity after complete infectious results is similar to that found in a previous study evaluating AE and IE (12). Another study showed a sensitivity of 90% and specificity of 83% at 2 weeks for the Graus possible AE criteria in adult patients with encephalitis, a much higher sensitivity and a lower specificity than seen in our study (16). As each phase of infectious testing may have differing times until results finalize (median 1.5 days and 4 days for PCR and infectious antibody testing, respectively, in our study), clinicians must make a decision on how thorough infectious testing must be to give a “reasonable exclusion” and initiate empiric immunotherapy for AE. These decisions are individualized for each patient based on presentation, severity, and individual, seasonal, and regional risk factors for IE. This study helps to inform such decisions and will ideally decrease the time from meeting criteria for AE to empiric immunotherapy—a median of 2.5–3 days of delay was seen in our study.

The Graus criteria for anti-NMDARE—the most common pediatric AE—had high specificity in our study, especially within the first few days after symptom onset. This criterion unfortunately showed a lower sensitivity compared to the general AE criteria over the first month—highlighting the utility of also using the symptom-based general possible AE criteria in screening for patients needing additional diagnostic evaluation. Compared to another study evaluating pediatric anti-NMDARE, our sensitivity was very close to theirs at early time points [week 1 (26 vs. 26%), week 2 (52 vs. 48%)], with slightly lower sensitivity (87 vs. 90%) and specificity (87 vs. 96%) at the final time point (17), and similar to that reported in adult patients (18).

A DEE score of 3 in our pediatric cohort gave a specificity of 100% for showing AE as being “highly unlikely” (i.e., in this context, for IE being likely), higher than the 75% seen in the training cohort of adult and pediatric patients with acute meningoencephalitis in the original paper (11). Sensitivity was much lower in our study however (29 vs. 92%). Differences between the two may be due to differences in identifying and defining our cohorts of IE and AE, or our focus on pediatric patients. Regardless, our study provides validation for use of this score in pediatric encephalitis—with a score of 3 giving a low probability of the encephalitis being autoimmune in origin.

### Symptoms and diagnostic testing

Our study elucidated potential areas for future refinements of the clinical criteria for AE in cohorts of encephalitis. Prior studies have found that compared to IE, AE is associated with higher rates of psychosis, movement disorders, non-convulsive or super-refractory status epilepticus, speech/behavior changes, and presence of CSF-restricted oligoclonal bands (OCBs), with lower rates of fever, headache, lower CSF nucleated cell counts and protein, and diffusion restriction on MRI-Brain (1, 5, 12, 19–24). In our study, we noted the presence of a personality or behavioral change, affective disorders, speech change, and sleep disorders to be more common in patients with AE. Fever, elevated CRP, elevated CSF protein, and an abnormal MRI were more common in IE. Sleep disturbance has not been reported as showing a significant difference between IE and AE previously, although this is a well-known clinical symptom in AE (25, 26).

From Figure 2 and Table 1, it is noted that in IE, if a second NP symptom was to present, it presented by at latest 3 days after initial NP symptom onset, while in AE, it could present up to 52 days after onset. Infectious causes of encephalitis have been noted in other studies to have a more acute progression than autoimmune causes (23, 27, 28). For constitutional symptoms, fever occurred in a much higher frequency in IE compared to AE—and in IE either occurred before or up to 2 days after initial NP symptom onset. In AE, only 16% had fever onset before/at Day 2. While prior studies have looked at temporal ranking and which symptom occurred first, second, etc. (29, 30), this study focused on temporal dispersion throughout a clinical course—allowing for assessment of unique temporal clustering of symptoms. These observations may indicate a useful and under-recognized feature in distinguishing AE and IE—the temporal clustering and temporal onset of symptoms.

The current AE criteria focus on differentiating AE from not only IE, but also primary psychiatric, epileptic, genetic, and neurodegenerative disorders, so sensitivity and specificity will vary greatly depending on the presenting symptom or clinical context they are applied to (encephalopathy, psychosis, seizure, encephalitis, etc.). In future clinical criteria for AE, it may be most useful to have criteria specific to the core presenting symptom—by limiting the potential mimics of AE that are being distinguished between, specific unique features of AE within that cohort may maximize accuracy of the clinical criteria model. For example, the APE^2^ and RITE^2^ scores have been developed specifically for evaluation of autoimmune epilepsy and encephalopathy and since been evaluated in other centers (31–33), and psychiatric symptom clustering has been evaluated for identification of autoimmune psychiatric disease (34).

In the same way, the development of specific criteria to distinguish between AE and IE is needed. The DEE score represents a step-forward in distinguishing rapidly between AE and IE, and this study provides validation for its use in pediatric patients. Our study supports the inclusion of fever and elevation in CSF protein within the DEE score, but also suggests evaluation of CRP, abnormal MRI-Brain, the presence of specific NP symptoms, and temporal onset of symptoms in future revisions of this criteria. Until new rapid technologies or novel biomarkers are developed (35–40), validated, and widely available, reliance on the clinical criteria discussed here and their refinements will remain a mainstay for empiric diagnosis and treatment, especially in resource limited settings.

### Limitations

Initial identification criteria for the study were chosen to ensure a broad catchment of the disease processes of interest. The strict and multi-layered exclusion criteria for both AE and IE were chosen to provide increased confidence in the final diagnosis, and to clearly differentiate disease processes for which immunotherapy may be beneficial or detrimental. However, by our strict criteria patients with true viral encephalitis (albeit etiology unconfirmed) or true antibody negative AE may have been excluded, weighting our data set away from that seen in clinical practice. Other studies have used broader “probable” AE and IE categories (12); we chose to focus on definite cases to decrease data variability and the risk of misclassification, but this decision also limited our study size. Additionally, we chose to exclude a very common cause of brainstem encephalitis at our institution, enterovirus A71, due to its distinctive features (41, 42). The majority of the cases occurred prior to the COVID-19 pandemic and the identification of post-COVID encephalopathy/encephalitis (43). It is not clear yet if future analysis of these cases will increase or decrease the sensitivity of the criteria tested here. It should also be noted that no cases of solely anti-MOG related AE were present in our study. On subsequent review, all patients with anti-MOG disease during the study time period presented with a demyelinating rather than encephalitic phenotype. As increased recognition of FLAIR-hyperintense lesions in anti-MOG-associated encephalitis with seizures (FLAMES) and other encephalitic phenotypes of anti-MOG disease occurs and testing increases (44), inclusion of these patients in future studies may alter the comparison of AE and IE clinical symptoms and testing from those given here. Finally, AE and IE may not be mutually exclusive—similar to NMDARE presenting after HSVE, post-infectious or para-infectious encephalitis may blur the lines between AE and IE. These cases were not considered in this study given the difficulty in categorizing these patients under the current AE criteria, and uncertainty regarding appropriate treatment approach.

For confidence in the diagnosis of antibody-negative AE, we applied the Cellucci criteria for pediatric antibody-negative AE—all patients who met Graus criteria also met Cellucci criteria. In our study, 28% of AE patients were antibody-negative after adjudication, and if only Cellucci criteria was applied, 59%, comparable to other pediatric studies (2–4). However, it may be that the Cellucci criteria does not accurately identify antibody-negative AE—our adjudicator AY identified several patients as AE that did not meet Cellucci criteria, and several patients meeting Cellucci criteria were adjudicated as another primary non-AE disorder. This lack of concordance points to the disconcerting notion that our knowledge of the spectrum and etiology of antibody-negative AE remains imperfect (10), and that even using objective clinical criteria or subjective expert clinical experience, we may be prone to under or overdiagnosis. While not the focus of this study, this topic by itself is worth of further evaluation—what factors clinicians consider the most in diagnosing antibody-negative AE, and if it is possible to capture and weight these factors objectively in a clinical criterion.

Finally, and importantly, this study was limited by its size, its location at a single center, and retrospective nature. The findings of this study will need to be confirmed in larger, multi-center prospective studies on pediatric encephalitis given these limitations.

## Conclusions

While the Cellucci and Graus criteria provide a guide for identification of pediatric AE, the sensitivity and specificity of these criteria do not increase above 90% within the first month of symptom onset in pediatric encephalitis. Rapid PCR testing provides the greatest contribution to ruling out infectious etiologies. A DEE score of 3 gives a low probability of AE in pediatric patients with encephalitis. Further refinement of criteria is needed to define antibody-negative AE and to improve early detection and treatment of pediatric AE within encephalitis presentations.

## Data availability statement

The raw data supporting the conclusions of this article will be made available by the authors, without undue reservation.

## Ethics statement

The studies involving human participants were reviewed and approved by Colorado Multiple Institutional Review Board (COMIRB), University of Colorado. Written informed consent from the participants' legal guardian/next of kin was not required to participate in this study in accordance with the national legislation and the institutional requirements.

## Author contributions

TP, RW, and RK contributed to data acquisition. TP and RK provided the statistical analysis and wrote the manuscript. All authors contributed to the design, implementation of the research, analysis and interpretation of the results, and review and revisions of the manuscript.

## Conflict of interest

Unrelated to this study: Author AP reports grants from University of Colorado and Rocky Mountain MS Center, consulting fees from Genentech/Roche and Alexion, and also receive honorarium from Medlink and publication royalties from Springer as co-editor of a textbook. Author RK reports grants from University of Colorado and Rocky Mountain MS Center and consulting fees from Sanofi-Genzyme. The remaining authors declare that the research was conducted in the absence of any commercial or financial relationships that could be construed as a potential conflict of interest.

## Publisher's note

All claims expressed in this article are solely those of the authors and do not necessarily represent those of their affiliated organizations, or those of the publisher, the editors and the reviewers. Any product that may be evaluated in this article, or claim that may be made by its manufacturer, is not guaranteed or endorsed by the publisher.

## Supplementary material

Supplementary Table 1 Clinical symptoms and paraclinical data in antibody-positive and antibody-negative AE.

Supplementary Figure 1 Sensitivity of the Cellucci and Graus criteria for pediatric **antibody-positive** AE over the first month after NP symptom onset utilizing **(A)** clinical symptoms alone (Symptoms Only), **(B)** both clinical symptoms and initial paraclinical diagnostic testing (EEG, MRI, CSF) without autoantibodies (Initial Diagnostics), and **(C)** assuming an idealized scenario where diagnostic testing are immediately obtained after meeting symptom criteria (Immediate Diagnostics).

Supplementary Figure 2 Sensitivity of the Cellucci and Graus criteria for pediatric **antibody-negative** AE over the first month after NP symptom onset utilizing **(A)** clinical symptoms alone (Symptoms Only), **(B)** both clinical symptoms and initial paraclinical diagnostic testing (EEG, MRI, CSF) without autoantibodies (Initial Diagnostics), and **(C)** assuming an idealized scenario where diagnostic testing are immediately obtained after meeting symptom criteria (Immediate Diagnostics).
