# Supplementary material for: Evaluation of multiple consensus criteria for autoimmune encephalitis and temporal analysis of symptoms in a pediatric encephalitis cohort
Source: Front Neurol. 2022 Sep 27;13:952317. doi: 10.3389/fneur.2022.952317 (PMC9552833; doi:10.3389/fneur.2022.952317)
Supplement: Supplementary Table 1 — Clinical symptoms and paraclinical data in antibody-positive and antibody-negative AE. [file Table_1.DOCX]

**Table S1.** Clinical symptoms and paraclinical data in antibody-positive and antibody-negative AE

|  | **Antibody-Positive AE (23)** | **Antibody-Negative AE**  **(9)** |
| --- | --- | --- |
| **Constitutional symptoms, n (%)** | | |
| Fever | 5 (22) | 3 (33) |
| Headache | 6 (26) | 5 (56) |
| Upper respiratory | 5 (22) | 4 (44) |
| Gastrointestinal | 8 (35) | 5 (56) |
| Myalgias | 0 (0) | 0 (0) |
| Rash | 2 (9) | 0 (0) |
| **Neuropsychiatric symptoms, n (%)** | | |
| Personality or behavioral change | 21 (91) | 8 (89) |
| Cognitive dysfunction or regression^1^ | 21 (91) | 9 (100) |
| Speech change | 21 (91) | 8 (89) |
| Seizure | 20 (87) | 4 (44) |
| Psychosis | 11 (48) | 4 (44) |
| Affective disorder | 12 (52) | 3 (33) |
| Dysautonomia | 6 (26) | 1 (11) |
| Movement disorder | 14 (61) | 3 (33) |
| Insomnia/hypersomnia | 11 (48) | 4 (44) |
| Focal neuro deficit^2^ | 15 (65) | 4 (44) |
| **Diagnostic abnormalities, n / no. tested (%)** | | |
| EEG *Any abnormality*  *Background slowing (generalized or focal)*  *Epileptiform discharges*  *Extreme delta brush* | 23/23 (100)  18 (78)  8 (35)  3 (13) | 9/9 (100)  7 (78)  3 (33)  0 (0) |
| MRI-Brain *Any abnormality*  *Meningeal enhancement*  *T2 hyperintensity, uni/bilateral temporal lobe*  *T2 hyperintensity, other grey/white matter* | 9/23 (39)  4 (13)  1 (3)  4 (13) | 6/9 (67)  0 (0)  1 (11)  1 (11) |
| CSF^3^  *Pleocytosis, >5 cells/µL*  *Pleocytosis, >20 cells/µL*  *Elevated protein, >45 mg/dL*  *Oligoclonal bands (≥2, CSF, unique)* | 14/23 (61)  5/23 (22)  2/22 (9)  11/14 (79) | 2/8 (25)  2/8 (25)  0/8 (0)  1/7 (14) |

| Blood *Elevated CRP*  *Elevated ESR* | 1/19 (5)  2/16 (13) | 0/8 (0)  1/6 (17) |
| --- | --- | --- |

| **Time until clinical criteria met** | | |
| --- | --- | --- |
| Symptoms only, days, median (range) | Graus: 5 (0-52)  Cellucci: 1 (0-52) | Graus: 2.5 (0-21)  Cellucci: 1 (0-28) |
| Full criteria, days,  median (range) | Graus: 9.5 (1-52)  Cellucci: 11 (1-72) | Graus: 7 (4-67)  Cellucci: 10 (1-68) |

^1^The symptom “cognitive dysfunction or regression” included those with cognitive dysfunction, altered mental status, memory change, or regression

^2^ Focal neurologic deficits included ataxia / gait imbalance, focal weakness, or cranial nerve palsies

^3^Traumatic CSF (>1,000 red blood cells / µL) was excluded for analysis of cell count and protein
