# Supplementary figures and images for: Evaluation of multiple consensus criteria for autoimmune encephalitis and temporal analysis of symptoms in a pediatric encephalitis cohort
Source: Front Neurol. 2022 Sep 27;13:952317. doi: 10.3389/fneur.2022.952317 (PMC9552833; doi:10.3389/fneur.2022.952317)

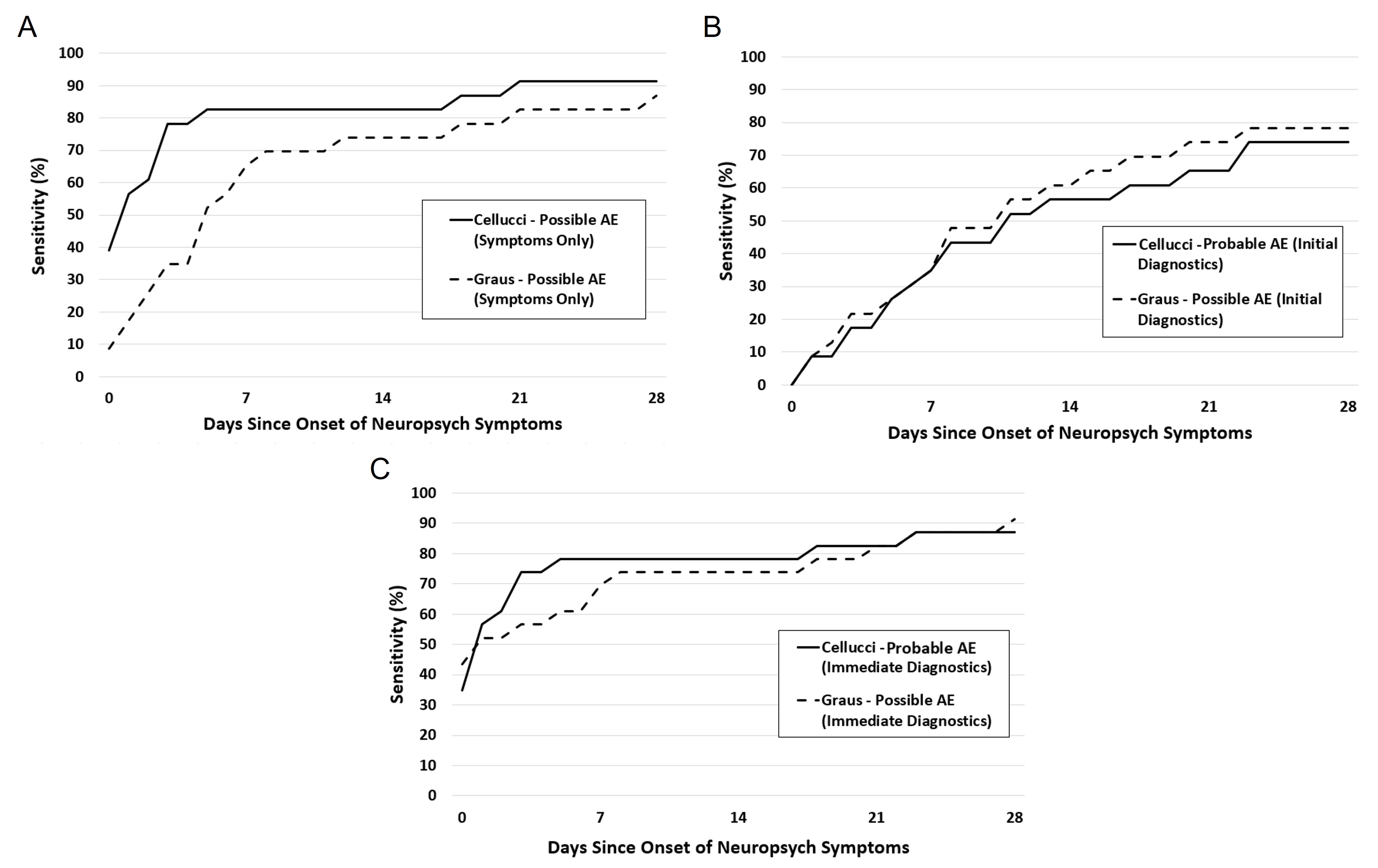

Supplement: Supplementary Figure 1 — Sensitivity of the Cellucci and Graus criteria for pediatric antibody-positive AE over the first month after NP symptom onset utilizing (A) clinical symptoms alone (Symptoms Only), (B) both clinical symptoms and initial paraclinical diagnostic testing (EEG, MRI, CSF) without autoantibodies (Initial Diagnostics), and (C) assuming an idealized scenario where diagnostic testing are immediately obtained after meeting symptom criteria (Immediate Diagnostics). [file Image_1.jpg]

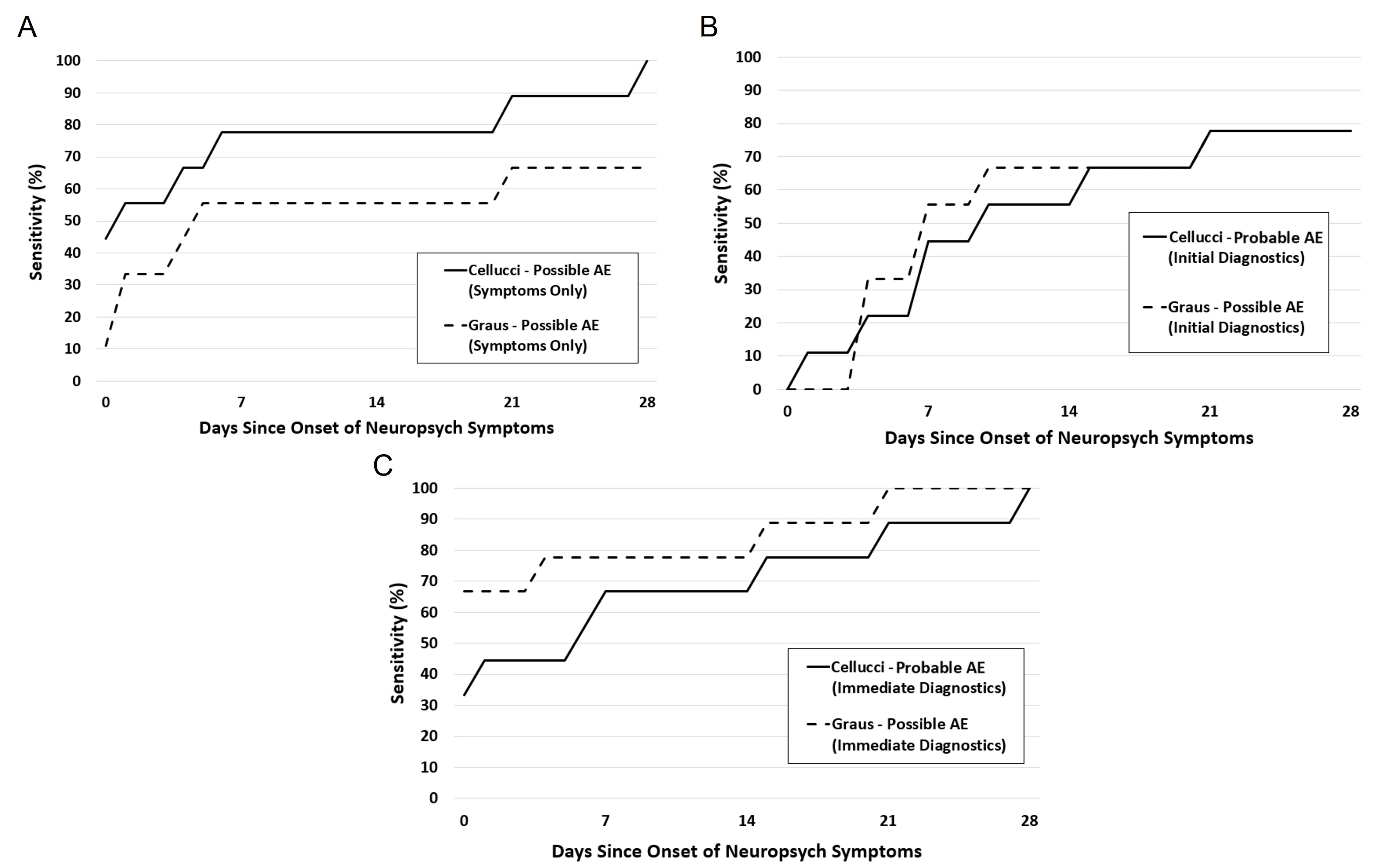

Supplement: Supplementary Figure 2 — Sensitivity of the Cellucci and Graus criteria for pediatric antibody-negative AE over the first month after NP symptom onset utilizing (A) clinical symptoms alone (Symptoms Only), (B) both clinical symptoms and initial paraclinical diagnostic testing (EEG, MRI, CSF) without autoantibodies (Initial Diagnostics), and (C) assuming an idealized scenario where diagnostic testing are immediately obtained after meeting symptom criteria (Immediate Diagnostics). [file Image_2.jpg]
